# Novel Continuous Fiber Bi-Matrix Composite 3-D Printing Technology

**DOI:** 10.3390/ma12183011

**Published:** 2019-09-17

**Authors:** Adi Adumitroaie, Fedor Antonov, Aleksey Khaziev, Andrey Azarov, Mikhail Golubev, Valery V. Vasiliev

**Affiliations:** 1 Analysis and Advanced Materials for Structural Design (AMADE), University of Girona, 17004 Catalonia, Spain; 2 Faculty of Mechanical Engineering and Design, Kaunas University of Technology, K. Donelaicio St. 73, LT-44029 Kaunas, Lithuania; 3 Anisoprint LLC, Skolkovo Innovation Center, 105005 Moscow, Russia; antonov@anisoprint.ru (F.A.); khaziev@anisoprint.ru (A.K.); golubev@anisoprint.ru (M.G.); 4 Department of Rocket and Space Composite Structures (SM-13), Bauman Moscow State Technical University, Ul. Baumanskaya 2-ya, 5, 105005 Moscow, Russia; 5 Ishlinsky Institute for Problems in Mechanics, Russian Academy of Sciences, 105005 Moscow, Russia; vvvas@dol.ru

**Keywords:** additive manufacturing, 3-D printing, fused filament fabrication, continuous fibers, polymer matrix composite, fiber reinforced polymer, thermoset, thermoplastic

## Abstract

A *new paradigm* in continuous fiber-reinforced polymer fused filament fabrication based on a thermoset-thermoplastic bi-matrix material system is proposed and proved. This totally new 3-D printing concept has the potential to overcome the drawbacks and to combine the advantages of separate thermoset and thermoplastic-based, fused filament fabrication methods and to advance continuous fiber-reinforced polymer 3-D printing toward higher mechanical performances of 3-D printed parts. The novel bi-matrix 3-D printing method and preliminary results related to the 3-D printed composite microstructure and performances are reported.

## 1. Introduction

Currently, there is an increasing demand for high-performance fiber-reinforced composite materials for structural applications in key industry sectors (e.g., aerospace and automotive). The common feature of these applications is the *lightweight design* strategy which triggers reduced structural weight while preserving high mechanical performances, less fuel consumption directly related to less carbon emission, and increased design flexibility compared to traditional isotropic materials. The concept especially applies to *fiber-reinforced polymers* (FRP) for structural application under severe loading conditions.

At the same time, digital *additive manufacturing* (AM, commonly referred to as *3-D printing*) has emerged as a relatively new and booming concept, a manufacturing method of extreme interest for further development and innovation due to its potential to bring complete modification of the production chain [1,2]: no need for complex tools and reduced need for auxiliary manufacturing systems, which translates into less associated costs, more efficient resource usage, and positive environment impact; adapted to low production rates yet at competitive costs; possibility to produce complex assemblies with fewer parts and fewer joining elements; flexibility to rapidly apply design changes, thus meeting the needs of a more and more dynamic market; almost zero manufacturing waste; and advanced human–machine interaction in a compact and predominantly computer-controlled environment for integrated design and manufacturing.

While AM itself is a wide field with different specific methods for different classes of materials [3,4], the particular material extrusion method of *fused filament fabrication* (FFF) is of special interest in regard to polymer 3-D printing. FFF methods for FRP composites are mostly dedicated to discontinuous short fiber reinforcement materials [3], which does not offer the high mechanical performances required for severe load applications. The most desirable properties and benefits of polymer matrix composites are achieved only for *continuous fiber-reinforced polymers* (CFRP). Research on methods for high-performance CFRP FFF is of high interest and rapidly developing in the USA [5,6,7,8], Europe [9,10,11,12], and Asia [13,14,15,16]. The most known name at the moment is the US-based company Markforged [8] whose solution allows for CFRP FFF based on Nylon (PA 6) matrix only, which is a considerable limitation in terms of material selection for various applications requirements. Overall, the current methods for high-performance CFRP FFF are still at the early stages of development, featuring a low degree of know-how and technological maturity.

Moreover, the main research and development trend for FRP FFF is based on *thermoplastic* (TP) polymer matrix for 3-D printed composite materials. The main disadvantage of TP-based technology resides in the high viscosity of the polymer melt which translates into low wettability and impregnation of the reinforcing fibers inside of the impregnation unit, which further translates into low adhesion bond strength between the fibers and the TP matrix, which eventually results into poor mechanical properties of the 3-D printed composite material. This is the reason why most of the current FRP FFF methods are based on a TP matrix that allows for a compromise between processability and the mechanical performances (e.g., ABS, PA, PLA, PC, and PS), which is a limiting factor for applications under high thermo-mechanical loading conditions. The higher the TP thermo-mechanical performances, the higher its melt viscosity [17] and the more difficult fiber impregnation and FFF processing are. Alternative FRP FFF methods based on fast photo-curable *thermoset* (TS) matrix, thus taking the TS processing advantage of low viscosity before curing and the consequent good wettability and impregnation of the fibers, are also under development [18,19]. However, the mechanical characteristics and performances of the photo-curable TS [20] are inferior to the TS used in high-performance engineering applications (e.g., thermo-curable epoxy).

On this background, the technology presented here aims to bring together the concepts of high-performance CFRP and FFF 3-D printing as the manufacturing method for composite parts. The FFF method was selected due to its ability to reproduce the main features and to recover the main advantages of the traditionally manufactured multidirectional laminates and was especially adapted to achieve the objective of producing high-performance CFRP composite parts.The presented research and development proves a **new paradigm** in CFRP FFF, based on a TS-TP bi-matrix material system, which would allow for overcoming the drawbacks and for combining the advantages of the separate TS and TP-based CFRP FFF approaches. The concept is based on the innovative idea and development of Anisoprint LLC, Russia [21,22]. The bi-matrix CFRP 3-D printing technology is described, and preliminary results related to the 3-D printed composite material microstructure and mechanical performances are presented. Directions for further investigation and development are identified.

## 2. Concept and Approach

**Figure 1:**
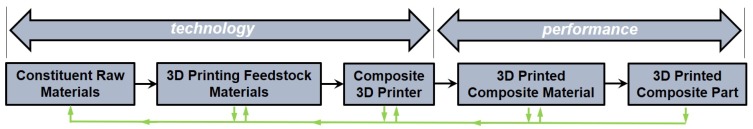
The systematic research and development approach.

The research aims to address the development of new materials, methods, and products in terms of multiple and coupled aspects (Figure 1): multiphase constituent raw input materials; development of new feedstock materials specially designed for specific 3-D printing methods; development of new 3-D printing technology to process feedstock materials and to output the composite material; properties and performances of the output composite materials and structural parts; and design guidelines for the new composite material and 3-D printing technology. The combination of the two main concepts of AM and CFRP brings a *technology–performance* double-focus of the research and development, which unfolds as an incremental–iterative system approach: incremental parts for the feedstock material development; composite 3-D printer development; and technology and products testing, verification, and validation and iterative corrective feedback provided from downstream to upstream increments (green line in Figure 1).

**Figure 2:**
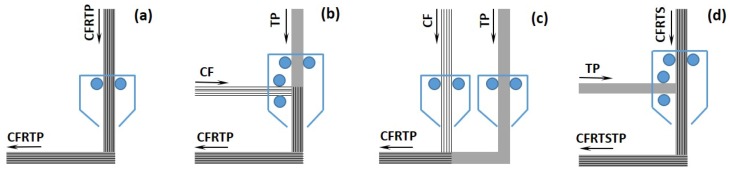
Methods for continuous fiber-reinforced polymer (CFRP) fused filament fabrication (FFF): (**a**) fibers embedded before the printer head; (**b**) fibers embedded inside of the printer head; (**c**) fibers added after the printer head; and (**d**) bi-matrix material system.

The core idea is the inclusion of *continuous* high-performance fiber reinforcements (e.g., carbon, glass, and aramid) into a high-performance polymer matrix filament, which is processed and extruded by the 3-D printer head, thus obtaining a CFRP composite material as the printer output. Current worldwide research methods for CFRP FFF investigate three possibilities of embedding continuous fiber reinforcement into the TP matrix extruded by the 3-D printer head (Figure 2):

(i) fibers are incorporated into the TP filament as a preprocessing step, outside of the 3-D printer; a *continuous fiber reinforced thermoplastic* (CFRTP) filament is thus pre-manufactured and can be stored, which is then fed to, processed by, and extruded by the 3-D printer head [8,9,16] (Figure 2a);
(ii) fibers and TP filament are separately fed to the 3-D printer head; fibers are incorporated into the TP melt inside of the printing head, and the resulting CFRTP melt is extruded by the 3-D printer head [13,14,15] (Figure 2b);
(iii) fibers and TP filament are separately fed to the 3-D printer; fibers are laid down on top of the extruded TP filament [10] (Figure 2c).

On this conceptual background, a completely innovative CFRP FFF approach, based on a TS-TP bi-matrix material system (Figure 2d) has been developed and is reported here:

(1) fibers are impregnated into the high-performance epoxy TS matrix, which is thermally cured as a pre-processing step, outside of the 3-D printer; a *continuous fiber-reinforced thermoset* (CFRTS) filament is thus pre-manufactured and can be stored;
(2) the CFRTS filament is fed to the composite 3-D printer together with a separate TP filament;
(3) the CFRTS filament is embedded into the TP melt inside of the printing head;
(4) the resulting *continuous fiber-reinforced thermoset-thermoplast* (CFRTSTP) composite filament is extruded by the 3-D printer head.

The bi-matrix TS-TP composite solution was first proposed by Vasiliev and Salov [23,24] without being initially related to 3-D printing technology; it was proposed for classical composite manufacturing processes and meant to combine the mechanical *performance* advantages and to mitigate the disadvantages of individual TS and TP-based composites. The concept of the TS-TP bi-matrix system was further investigated and developed by Anisoprint in conjunction with the idea of CFRP FFF [21,22,25], now also meant to combine the *processing* advantages and to mitigate the disadvantages associated with FFF 3-D printing technologies based on either TP or TS polymer matrixes.

The bi-matrix FFF 3-D printing concept is based on the following:

– the *fact* that the TS matrix offers the needed *processability* characteristics (especially low viscosity to ensure a good impregnation of the reinforcing fibers) to obtain a good quality and high-performance CFRTS composite filament (as it is demonstrated by current classical CFRTS composite manufacturing methods).
– the *fact* that the TP matrix offers the needed *processability* characteristics for the FFF method: enough viscosity of the melt phase to be extruded by the 3-D printer head and optimal solidification time during cool down phase in order to ensure maintenance of the desired shape of the 3-D printed part without self-weight distortions (as it is demonstrated by current commercial TP FFF systems).
– the *assumption* that the good FFF *processability* characteristics can be preserved for the multi-material fiber TS-TP composite system.
– the *assumption* that a good adhesion bond strength can be achieved between the constituents of the multi-material system in order to ensure high *performance* of the 3-D printed composite.

The core advantage of the TS-TP bi-matrix CFRP FFF method is the *uncoupling* of the fibers impregnation and extrusion steps of the composite 3-D printing process: *impregnation* is realized outside of the 3-D printer, as a separate and individually well-controlled pre-manufacturing step based on a high-performance TS polymer matrix (i.e., thermo-curable epoxy), thus obtaining a pre-manufactured CFRTS filament; *extrusion* is realized by the 3-D printer head by embedding the CFRTS filament into the TP melt (co-extrusion), thus obtaining a high-performance CFRTSTP output filament. In this way, one of the main limitations of other current CFRP FFF methods and systems, namely the poor impregnation of the reinforcing fibers by the high-viscosity TP matrix, is released by designing the composite 3-D printing process in this uncoupled manner. Moreover, due to its impregnation-extrusion uncoupling feature, the bi-matrix 3-D printing technology is also amenable to using engineering-graded high-performance TP polymers (e.g., PEEK and PEI) [17] for the CFRP FFF process, which is another strong challenge for other CFRP 3-D printing methods.

## 3. Materials and Method

**Figure 3:**
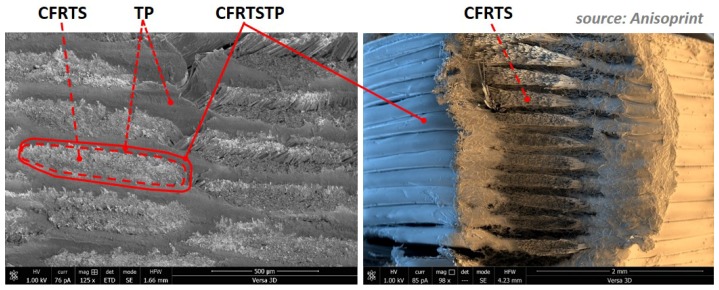
Three-dimensionally printed CFRP composite material based on the bi-matrix thermoset (TS)-thermoplastic (TP) FFF technology.

The TS-TP bi-matrix composite FFF reported here is possible thanks to fine tuning and matching the extruder operating temperature to the TP melting temperature (Tm) and to the TS glass transition temperature (Tg) [21]. This way, the inherent stiff and brittle pre-cured CFRTS filament softens above TS Tg and can be processed through the printer head and extruded together with the TP melt. Moreover, softening of the cured TS matrix also allows for composite material consolidation during 3-D printing layup, thus obtaining a high-quality void-free 3-D printed composite (Figure 3). This is possible by the special design of the Anisoprint extruder nozzle [22], which applies pressure upon extrusion, thus consolidating the last deposited filament on top of the previous ones. This is the reason why the initial round CFRTSTP composite filament (according to the round profile of the extrusion nozzle) takes the elliptical shape shown in Figure 3. In this way, an adjustable layer thickness (min. 0.06 mm) can be obtained based on a nozzle diameter of 0.9 mm by adjusting the extruder nozzle consolidation pressure. A layer thickness of about 0.32 mm corresponds to the 3-D printed composite material in Figure 3.

**Figure 4:**
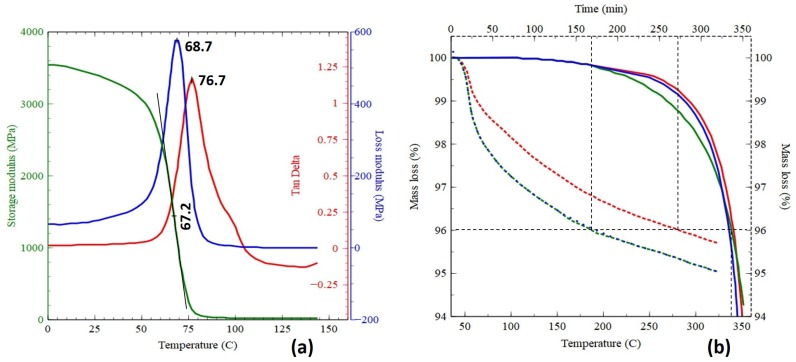
Thermoset (TS) matrix characteristics: (**a**) Dynamic mechanical analysis (DMA) results; (**b**) Thermal gravimetric analysis (TGA) results.

Key material factors for the bi-matrix composite 3-D printing technology are the TS formulation and properties as well as TS impregnation of the dry reinforcing fibers and TS curing process; TP formulation and properties as well as TP melting process; the CFRTS/TP co-extrusion process; and adhesion bond strength between the material system interfaces (i.e., fibers/TS, TS/TP, and TP/TP). The TS matrix is an in-house modified epoxy formulation (Anisoprint proprietary) featuring Tg = 67 ${}^{\circ }$C (The dynamic mechanical analysis (DMA) results are presented in Figure 4a). The TP matrix for the results reported here is PLA [26] featuring Tm = 180 ${}^{\circ }$C. Reinforcing carbon fiber filaments [27] of three tow-numbers (3K, 1.5K, and 1K) have been investigated for the presented composite 3-D printing technology. Currently, preference was given to the 1K filament due to higher flexibility of the corresponding CFRTS filament, which is needed to store the pre-manufactured CFRTS filament on FFF filament roles without bending-induced damage.

Three-dimensional printing parameters are also essential to achieving good properties of 3-D printed composite materials. Thus, a printing (nozzle) temperature of 210 ${}^{\circ }$C and a printing speed of 200 mm/min were applied for the given material system in order to ensure good material compaction, as well as good TS/TP and TP/TP adhesion. According to the composite extruder design, the CFRTS filament is exposed for 6 s to above the Tg printing temperature, which is enough to acquire the necessary TS softening without degradation. According to thermal gravimetric analysis (TGA) results (Figure 4b), significant degradation (4% mass loss) of the TS matrix only occurs around 340 ${}^{\circ }$C for short time exposures (solid curves) and over 3–5 h for long time exposures at 250 ${}^{\circ }$C (dotted curves).

**Figure 5:**
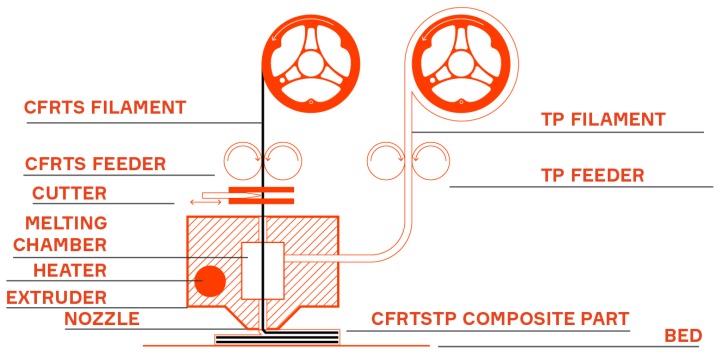
Continuous fiber-reinforced thermoset-thermoplast (CFRTSTP) composite 3-D printer schematic.

**Figure 6:**
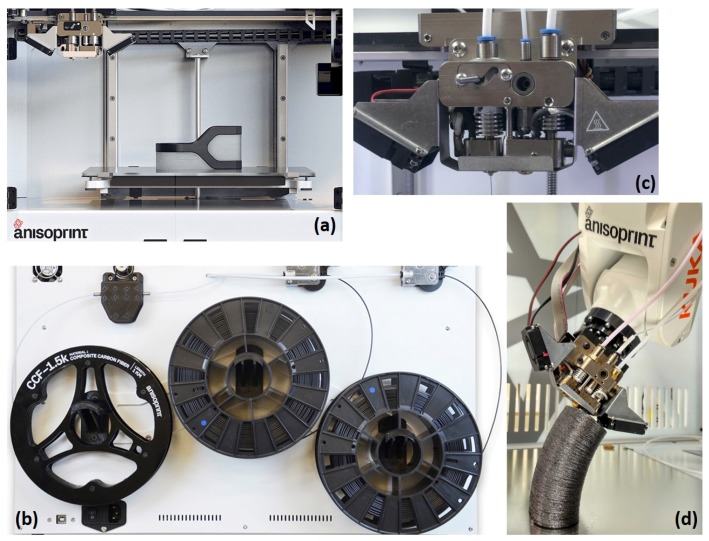
CFRTSTP composite 3-D printer design: (**a**) front view: 3-D printing volume; (**b**) back view: feedstock filaments roles; (**c**) composite extrusion head; and (**d**) extrusion head on a 5-axis robotic arm.

A schematic of the FFF CFRTSTP 3-D printer is presented in Figure 5. The CFRTSTP printer design corresponding to the current development stage is shown in Figure 6. The feedstock filament roles can be seen in Figure 6b: one for the CFRTS filament, one for the neat TP filament to be co-extruded with the CFRTS filament, and one for the neat TP filament to be used either as 3-D printing infill or support material if needed. Two FFF extrusion nozzles can be seen in Figure 6c: one for the CFRTSTP extrusion and one for the infill/support TP extrusion. The infill/support TP can be the same or different from the TP used for the CFRTSTP filament.

## 4. Results and Conclusions

SEM images of the material microstructure are presented in Figure 3. Mechanical properties of the CFRTSTP composite (T-300 carbon fibers, epoxy TS, and PLA TP) based on the bi-matrix FFF 3-D printing method described here are presented in Table 1. For direct comparison with high-performance composites manufactured through classical methods, the 3-D printed CFRTSTP reported here corresponds to a full density unidirectional infill pattern. For comparison, reported values [28,29,30] for the CFRTP composite based on Markforged technology (T-300 carbon fibers and PA6 TP matrix) of the same infill density and pattern are also presented in Table 1. Reference values [31] for CFRP composites based on the classical manufacturing method of unidirectional prepreg sheet hot compression molding (T-700 carbon fibers and separate epoxy TS and PA6 TP matrix) are shown in Table 1 as well. Due to the fact that no data could be found for the classically manufactured composite system T-300/PA6, attention should be paid when comparing the mechanical parameters in Table 1 to the different mechanical properties of the T-300 (E = 230 GPa, X = 3530 MPa) and T-700 (E = 230 GPa, X = 4902 MPa) carbon fibers reinforcements [27].

It can be inferred from the data in Table 1 that, even at lower fiber volume fractions, the novel CFRTSTP composite 3-D printing technology competes well and even takes the lead compared to the market leader Markforged in terms of both tensile and compressive properties. This can be explained based on better impregnation, support, and stress transfer mechanisms offered by the TS matrix to the reinforcing fibers inside of the CFRTSTP material, which is the core of CFRTSTP 3-D printing technology.

A high-fiber volume fraction (≈60%) and corresponding high mechanical characteristics (E ≈ 140 GPa, X ≈ 1950 MPa) can be achieved for the preprocessed CFRTS filament. However, the overall fiber volume fraction and mechanical characteristics of the 3-D printed CFRTSTP material are reduced by the addition of the TP matrix (≈25–27% overall fiber volume fraction is currently achieved). Further improvement of the mechanical performances can be expected through further technology refinement, which would allow for a reduction in the TP layer thickness between two adjacent CFRTSTP filaments (Figure 3) and, accordingly, for an increase in the CFRTSTP filament fiber volume fraction towards ≈35%, which would bring a corresponding increase of the stiffness and strength mechanical characteristics of the FFF 3-D printed CFRTSTP composite.

**Figure 7:**
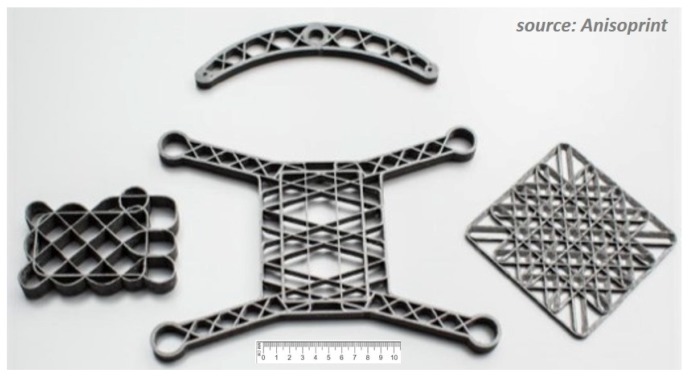
Three-dimensionally printed CFRP composite structural parts based on bi-matrix TS-TP FFF technology.

It is thus envisioned that the 3-D printed CFRTSTP composite parts cannot fully compete in terms of the maximum achievable mechanical properties against the high-performance TS or TP composites manufactured by the classical methods (where the fiber volume fraction can reach 65–70%). However, it is nevertheless expected that the composite parts obtained through the CFRTSTP FFF method presented here will benefit from (i) the inherent high flexibility of 3-D printing technology, allowing for manufacturing of composite parts not easily obtained through classical manufacturing methods (see Figure 7); (ii) combined *processing* advantages of CFRP FFF technologies based on separate TS and TP matrix systems; and (iii) combined *performance* advantages of the TS-TP bi-matrix composite system (e.g., shifting the sudden brittle failure mode specific to TS composites, which is one of the main drawbacks of TS-based composites operating under severe loading conditions, toward the more progressive ductile failure specific to TP composite [32,33]).

In terms of CFRTSTP structural parts applications [34,35], these are dimensionally limited by the build volume of the 3-D printer (Figure 6a and Figure 7). Examples include composite brackets, connectors, lattice structures, drone bodies, cubesats, and any other kind of small–medium-dimension and complex 3-D geometry composite parts. However, the method is amenable to being extended to robotic arm manipulation of the composite 3-D printing head, which would thus allow for scale up of the composite part dimensions (Figure 6d).

These preliminary results show the validity and the potential of the novel CFRP 3-D printing technology based on the TS-TP bi-matrix FFF concept. Work in progress as well as directions of further research and development in order to improve the technology and to increase the performances of the CFRP 3-D printed composites include the following:

– to achieve an increased fiber volume fraction of the CFRTSTP composite filament;
– to upgrade to using the 1.5K carbon fiber tow, which shows acceptable flexibility and is more affordable than the 1K fiber tow;
– to upgrade to using high-performance engineering polymers (e.g., PEEK and PEI) for the TP matrix;
– to deploy advanced polymer science and nanotechnology in order to optimize the processability and performance characteristics of the feedstock materials;
– to deploy a comprehensive material evaluation and testing program (including destructive and nondestructive material characterization, as well as virtual simulation of the 3-D printed composite mechanical behavior) in order to provide both better feedback to the material development and material performance metrics databases;
– to provide composite parts design guidelines to consider the influences of the FFF processing parameters and the bi-matrix composite microstructure.

The upgrade to high-performance TP matrices (e.g., PEEK and PEI) is considered of high interest for structural applications under severe thermo-mechanical loading conditions, and the research and development team is actively pursuing this objective. However, this task also requires reformulation of the TS matrix, which is due to the high Tm of high-performance TP (e.g., 343 ${}^{\circ }$C for PEEK [26]) value that takes the TS matrix into the degradation range shown by TGA (Figure 4b). Thus, a special high-temperature epoxy formulation or even replacing the epoxy with a more temperature-resistant TS (e.g., bismaleimide) is needed.

## Figures and Tables

**Table 1:** Mechanical properties of carbon CFRP.

Material	Density	Fiber Vol. Fraction	Elastic Modulus	Tensile Strength	Compressive Strength
	(g/cm${}^{3}$)	(%)	(GPa)	(MPa)	(MPa)
CFRTSTP *	**1.33**	**25–27**	**60**	**750**	**290**
CFRTP * [28,29,30]	1.40	27–40	53–68	667–719	223
CFRTP ** [31]	1.45	42	98	1309	–
CFRTS ** [31]	1.58	46	109	1664	–

* 3-D printing. ** classical manufacturing.

## Abbreviations

The following abbreviations are used in this manuscript:

AM	additive manufacturing
FFF	fused filament fabrication
FRP	fiber reinforced polymers
CFRP	continuous fiber reinforced polymers
TP	thermoplastic
TS	thermoset
CFRTP	continuous fiber reinforced thermoplastic
CFRTS	continuous fiber reinforced thermoset
CFRTSTP	continuous fiber reinforced thermoset-thermoplast
DMA	dynamic mechanical analysis
TGA	thermal gravimetric analysis
